# The Strong Light‐Emission Materials in the Aggregated State: What Happens from a Single Molecule to the Collective Group

**DOI:** 10.1002/advs.201600484

**Published:** 2017-02-21

**Authors:** Qianqian Li, Zhen Li

**Affiliations:** ^1^ Department of Chemistry Hubei Key Lab on Organic and Polymeric Opto‐Electronic Materials Wuhan University Wuhan 430072 China

**Keywords:** aggregated states, aggregation‐induced emission, light‐emission materials, molecular packing modes, room‐temperature phosphorescence

## Abstract

The strong light emission of organic luminogens in the aggregated state is essential to their applications as optoelectronic materials with good performance. In this review, with respect to the aggregation‐induced emission and room‐temperature phosphorescence luminogens, the important role of molecular packing modes is highlighted. As demonstrated in the selected examples, the molecular packing status in the aggregate state is affected by many factors, including the molecular configurations, the inherent electronic properties, the special functional groups, and so on. With the consideration of all these parameters, the strong fluorescence and phosphorescence in the aggregated state could be achieved in the rationally designed organic luminogens, providing some guidance for the further development.

## 1 Introduction

Organic light‐emitting materials, generally bearing the π‐conjugated structures, have been a hot topic for their wide applications, such as data storage, photoswitches, organic light‐emitting diodes (OLEDs), organic light‐emitting transistor (OLET) devices, and sensors.^1^, ^2^, ^3^, ^4^, ^5^, ^6^, ^7^, ^8^, ^9^, ^10^, ^11^, ^12^ Since the performance heavily depends on the inherent physicochemical properties of materials, especially the emitting efficiency in solid state, it is badly needed to develop excellent luminogens with strong emissions in the aggregated state. Actually, most of the π‐conjugated molecules demonstrate strong emissions in dilute solutions (almost in the single molecule state), but partially or even completely quenched emission in the solid state. This phenomenon is mainly ascribed to the intense intermolecular π–π stacking interactions with the famous name of the concentration quenching effect termed more than half a century ago,^13^ rather than their inherent electronic structures. And the noncovalent intermolecular interactions are considered to be determined by the distances among adjacent molecules and their packing status.

**Scheme 1:**
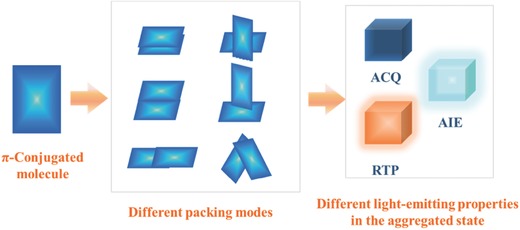
The different emissive behaviors by various packing modes of π‐conjugated molecules. **ACQ**: aggregation‐caused quenching, **AIE**: aggregation‐induced emission, **RTP**: room‐temperature phosphorescence..

**Scheme 2:**
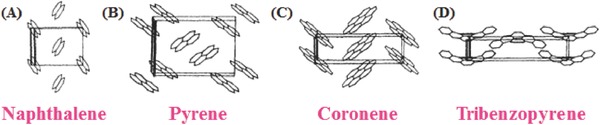
The crystal packings of basic aromatic rings: A) herringbone, B) sandwich herringbone, C) γ‐structure, and D) β‐structure. Definition: in the herringbone structure, the nearest neighbors are nonparallel, and the distance along the short axis (*D*_sa_) is larger than 5.4 Å, and shorter than 8.0 Å; in the sandwich herringbone packing, the herring‐bone motif is made up of sandwich‐like dimers, and *D*_sa_ is above 8.0 Å; in γ‐structure, the main interactions are among the parallel adjacent molecules, and *D*_sa_ is in the range of 4.6–5.4 Å; the β‐structure is characterized by “graphitic” planes, and *D*_sa_ is below 4.2 Å.

So far, as reported in literature, for the π‐system in solid state, there are mainly four kinds of intermolecular interactions: electrostatic interactions, dispersion interactions, attractive and repulsive orbital interactions, and hydrogen bonding interactions.^14^, ^15^, ^16^ Nevertheless, as to what extent these components contribute to the final packing modes of π‐conjugated molecules (**Scheme** **1**), it is still a matter of debate, possibly due to the diversity of the spatially anisotropic nature of the aromatic rings. While small arenes, such as benzene, naphthalene, and anthracene, display herringbone stacking modes, the larger ones are liable to pack as sandwich herringbone, γ‐ or β‐structures (**Scheme** **2**), according to the classification of Desiraju and Gavezzotti.^17^, ^18^ However, as to more complicated π‐conjugated molecules, it is very hard to predict the π‐stacking modes, just by analyzing different intermolecular interactions. Thus, the single crystal structure is very important for the detailed investigation of different packing modes. Actually, sometimes, with special packing modes, some luminogens could exhibit unexpected excited properties, much different from their behaviors in solutions, indicating the vital role of molecular packing and intermolecular interactions. Taking the current two hot topics of aggregation‐induced emission (AIE) and room‐temperature phosphorescence (RTP) as the typical examples, the performance of luminogens is mainly determined by both their inherent electronic properties and packing status in solid state. Thus, in addition to the traditional exploration of the structure–property relationship, the influence of the molecular packing, in solid state, should be seriously considered, as partially disclosed by many scientists in their published papers.^19^, ^20^, ^21^, ^22^, ^23^ In this paper, with some elaborately selected examples, we would like to discuss the possible relevance between the molecular structures and packing modes, and the related influence on the performance, with the aim to bridge the gap between the single molecule and the corresponding emission property in solid state.

## 2 The Aggregation‐Induced Emission Luminogens (AIEgens)

**Figure 1:**
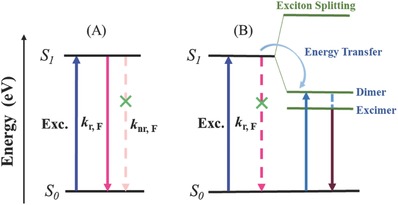
Energy‐level diagram of the relevant photophysical processes in AIE materials.

AIEgens are a special class of luminogens, completely opposite to aggregation‐caused quenching (ACQ) ones. Their emissions are much more brilliant in the practically useful solid state than in solution, exhibiting the academic value and practical applications in life science and biomedical engineering.^24^, ^25^, ^26^, ^27^, ^28^, ^29^ Basically, the emission of AIEgens in the aggregated state can be derived from two ways with different packing modes. In most cases, AIE is realized by the restriction of intramolecular motions (RIM) in the single molecule state, which blocks the nonradiative pathway for the excitons to decay (*k*_nr,F_ in **Figure** **1**A), with the activated radiative transition (*k*_r,F_ in Figure 1A).^30^ The structural feature of these AIEgens is the highly twisted configuration, to avoid the possible intermolecular π–π interactions. However, some planar conjugated molecules also exhibit AIE characteristic with the high‐efficiency excimer fluorescence from the pairwise (or sandwich herringbone) stacking state (Figure 1B).^31^ The excimer emission with the red‐shifted fluorescent spectrum comes from an excited‐state molecule close to another ground‐state molecule, or the formed dimer in the ground state. For example, pyrene exhibits weak emission at 350–400 nm in solution, but much stronger fluorescence peaked at about 480 nm, indicating the formation of intramolecular excimers.^32^

Thus, the essential features of AIE materials are complicated and hard to be controlled accurately, which are not only related to the configuration and electronic property of the single molecule, but also the packing modes with different types. After analyzing the molecular structures of many AIE materials in detail, some possible influence factors to the fluorescence in solid state are summarized and listed as follows, including the different sizes of the aromatic rings, the linkage positions of the substituent moieties, and so on.

### 2.1 The Sizes of the Aromatic Rings

**Figure 2:**
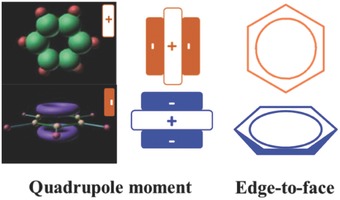
The packing mode of benzene in the aggregate state.

As to the AIEgens with the inherent mechanism of RIM, generally, there are some aromatic rings acting as rotors, and the phenyl ring is the most frequently presented one. As shown in **Figure** **2**, in solid state, the charges of benzene on the atoms can be set from the observed quadrupole moment. Positively charged hydrogens on the edge of one benzene plane, for example, the one in yellow, would be attracted, because of the natural Coulombic force, by negatively charged carbons of adjacent benzene molecules (in blue color), leading to the herringbone packing in its crystal and the resultant edge‐to‐face orientation of molecular dimer.^33^, ^34^, ^35^ This stacking mode could suppress the strong π–π stacking interactions of the mother luminogens, contributing much to the AIE characteristic.

**Figure 3:**
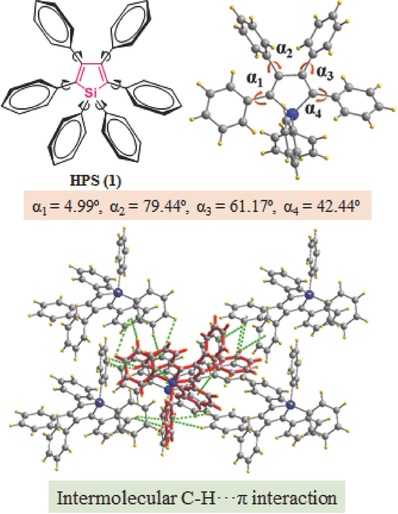
Chemical structure of HPS and its packing modes in the crystal. The crystal structures are retrieved free of charge from the Cambridge Crystallographic Centre (CCDC‐195948).

For example, in the famous AIEgen of hexaphenylsilole (HPS) (**Figure** **3**), six peripheral phenyl groups help to avoid a dense face‐to‐face packing structure, but a more loose one without π–π stacking interactions in the solid state.^36^ Also, its propeller‐like conformation with the large torsion angles (up to 79.44°) between the peripheral phenyl rings and the central silacyclopentadiene or silole plane can suppress the strong intermolecular interactions in a large degree, as confirmed by the long distances (9.363–10.043 Å) between the silole cores in the unit cell of HPS crystal. Alternatively, massive intermolecular C—H⋯π interactions exist between the adjacent HPS molecules, which help to restrict the free rotational motions of the phenyl rings, unlike the case in dilute solution. Thus, the dilute acetone solution of HPS is nonemissive, with a negligibly low fluorescent quantum yield (Φ_F_ ≈ 0.1%), but dramatically enhanced Φ_F_ (≈220‐fold) in the aggregated state or solid state is observed, as the result of the blocked nonradiative channel.

**Figure 4:**
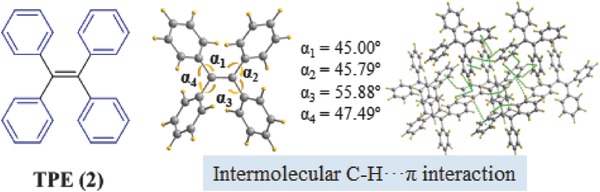
Chemical structure of TPE and its packing modes in the crystal. The crystal structures are retrieved free of charge from the Cambridge Crystallographic Centre (CCDC‐1275289).

Similar case could be found in another star molecule of AIEgens, tetraphenylethylene (TPE).^37^, ^38^, ^39^, ^40^ As shown in **Figure** **4**, in TPE, the central olefin stator is surrounded by four phenyl rings with the dihedral angle in the range of 45°–55°. In solution, the dynamic rotations of the phenyl rings around the single‐bond axes dissipate the exciton energy nonradiatively, making TPE nearly nonemissive. Once aggregated, the highly twisted molecular conformation and the electronic nature of phenyl rings as the peripheral units, result in the massive intermolecular C—H⋯π interactions, which can restrict the intramolecular rotation of phenyl rings in a large degree, directly leading to the strong light emission in the solid state. However, if the peripheral units of TPE were replaced by other aromatic rings, for example, naphthalene ring, the packing modes of the corresponding molecules in the solid state varied much for the varied electronic property and conjugated plane. In naphthalene, it is well established that the α and β‐positions have very different reactivity, probably due to the different net atomic charges as partially demonstrated by the calculated wavefunction of the molecule (ab initio quantum mechanics). Thus, the edge‐to‐face interaction, present in the benzene crystal, might become somewhat less important for naphthalene, alternatively, the dispersion interaction played a more important role.

**Figure 5:**
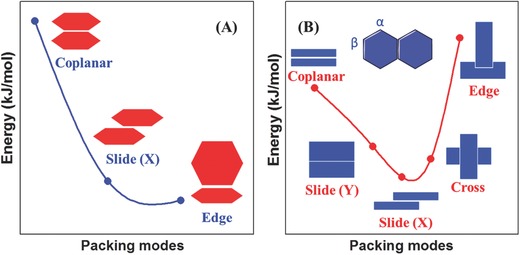
The possible stacking modes in the dimer of benzene and naphthalene and the corresponding minimum energies.

To understand spatial molecular relationships in the dimer models of benzene and naphthalene deeply, several constrained orientations were considered (**Figure** **5**).^41^ The reference molecule was placed in *XY*‐plane with its long axis along *X*. In the coplanar model, the second molecule is placed on the top at the distance *Z*; in the cross model, the molecule on top rotates 90° around an axis between the molecular center; in the edge model, one molecule can be perpendicular to another. In addition, the top molecule can be allowed to slide along *X* or *Y*. The intermolecular energies of these models were computed for different distances between molecular centers, and the minimum‐energy structures obtained for the models are shown in Figure 5. The edge model is the best one for benzene, even though the distance between molecular centers is larger with the small dispersion interaction, in a good agreement with the crystal structure. As to the naphthalene ring, the energy of the edge model is higher than that of the coplanar one, suggesting that a strict edge orientation is not preferred in the naphthalene dimer. Probably, an intermediate model will be the optimum, with a structure between the edge and coplanar models.

**Figure 6:**
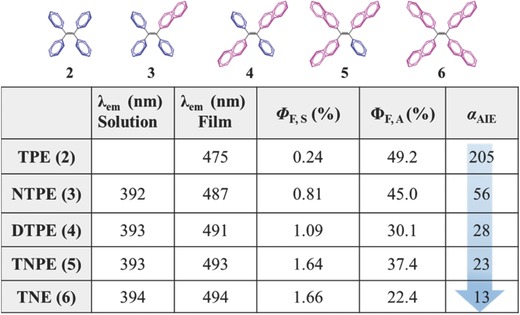
Systematically replacing the phenyl rings of TPE with naphthalene and the corresponding changes of different optoelectronic parameters.

Thus, from TPE to TNE (**Figure** **6**), with the increased number of naphthalene moieties as rotors, the emission enhancement factor (α_AIE_, which is defined as fluorescence quantum yield in the aggregate state (Φ_F,A_) divided by the fluorescence quantum yield in the solution state (Φ_F,S_)), decreases gradually from 205 to 13, mainly due to the different alignments of the benzene and naphthalene moieties in the solid state, in which, the larger size of the naphthyl unit is more prone to form the intermolecular π−π stacking, giving rise to intense intermolecular interactions.^42^ Thus, the Φ_F,A_ decreases with the continual replacement of phenyl rings by naphthyl units. Meanwhile, the ability of the larger naphthyl group to dissipate energy becomes weaker, as exemplified by the slight raise in Φ_F,S_ with the increased number of naphthyl groups. This systematic work suggests that the size of the peripheral moieties is extremely important to the AIE activities and the fluorescent properties in the solid state.

**Figure 7:**
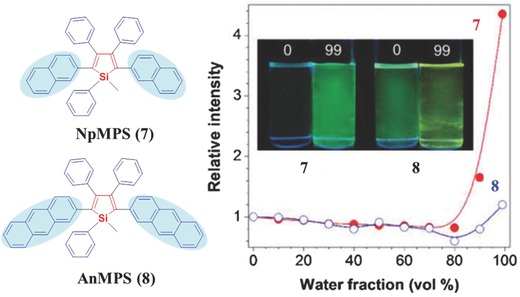
Chemical structures of compounds **7** and **8** and plots of their fluorescence intensity versus water faction in THF/water mixtures. Inset: Fluorescent photographs of **7** and **8** in THF/water mixtures (*f*_w_ = 0 and 99 vol%).

Further enlarging the size of peripheral moieties from naphthalene to anthracene, the coplanar model with the intramolecular π–π stacking is even more suitable by calculations, which can decrease Φ_F_'s of the resultant molecules in the aggregated state. As shown in **Figure** **7**, the Φ_F_ value of **7** with naphthyl moieties at the 2,5‐positions of silole is 37.0% in the film state, however, once the two naphthyl groups were replaced by anthracyl ones, compound **8** shows a film‐state Φ_F_ of 14.0%.^43^ The similar trend can be obviously observed from their fluorescent intensity versus water faction in tetrahydrofuran (THF)/water mixtures. Thus, does it mean that the larger sizes of aromatic rings can lead to the fluorescence quench effect, then to the weak AIE activity? Not exactly, pyrene with even larger size exhibits strong AIE property for the formation of excimers.

**Figure 8:**
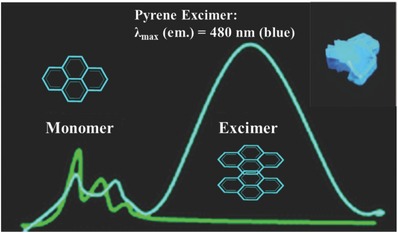
The packing mode of pyrene and the strong fluorescence from excimer.

The structure of the pyrene crystal is one composed of partially overlapping card‐packed dimeric units, in which, the pyrene molecules of each pair with coplanar mode are separated by a distance of 3.53 Å (**Figure** **8**).^32^ When a pyrene molecule in the crystal absorbs light, two parallel molecules will tend to move toward one another, to form the more complete overlap. As a result, a red‐shifted and broad emission band of the excimer in the range of 450–500 nm could be observed with strong fluorescence, much different from the weak emission of the single pyrene molecule in the UV region. Thus, the strong π–π stacking with the pairwise alignment can also be beneficial to the enhancement of fluorescence in the aggregated state. Similar to this packing mode, more emissive excimers have been reported, and the emission spectra can extend to the red or even far‐red region, showing the potentially application in biology field as fluorescent probes.^44^, ^45^, ^46^, ^47^, ^48^

From benzene to pyrene, the enlarged sizes of the aromatic rings favor the coplanar packing mode with the shortest distance between the molecular centers, mainly due to the dispersion attraction of the adjacent molecules. Normally, this strong π–π stacking can quench the fluorescence in the aggregated state, but the formed pyrene excimer exhibits the opposite effect, indicating the importance of the alignment in the aggregated state. For the AIEgens with RIM mechanism, the edge‐to‐face orientation, which is favored by the electrostatic interaction between the adjacent molecules, plays a significant role for the fluorescence in solid state.

### 2.2 The Linkage Positions of the Substituent Moieties

**Figure 9:**
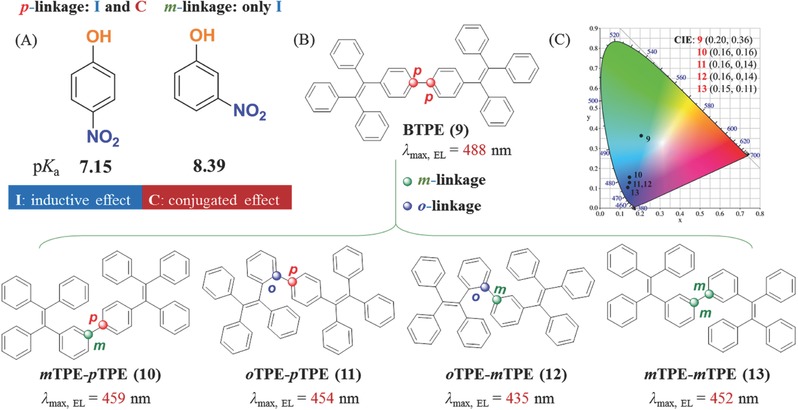
A) The basic knowledge of *p*‐nitrophenol and *m*‐nitrophenol; B) chemical structures of **9**–**13** with different linkage modes; C) the CIE coordinates of **9**–**13**.

In the textbook of basic organic chemistry, the different positions of nitro and hydroxyl groups in nitrophenols (**Figure** **9**A), affect their p*K*_a_ values for the varied inductive and conjugation effect. Accordingly, the different linkage positions of the substituent moieties in AIEgens, especially the conjugated rings and functional groups, result in different geometry configurations, optoelectronic properties and stacking modes in the aggregated state.^49^, ^50^, ^51^, ^52^, ^53^ For example, by utilizing the different conjugation effect of *ortho*‐, *meta*‐, and *para*‐linkages of two TPE units, the resultant compounds **9**–**13 (**Figure 9B) exhibited different electroluminescence spectra with the maximum electroluminescence wavelength (λ_max_) in the range of 435–488 nm. The Commission Internationale de l'éclairage (CIE) chromaticity coordinates (Figure 9C) also show that the *ortho*‐ and *meta*‐linkages can weaken the conjugation effect of the two TPE moieties with deep‐blue emissions of **10**–**13** and the sky‐blue emission of **9**, mainly due to their different geometry configurations. From the single molecule to the aggregated state, these varied geometry configurations can result in different intermolecular interactions of the adjacent molecules and packing modes in the crystal structures.

**Figure 10:**
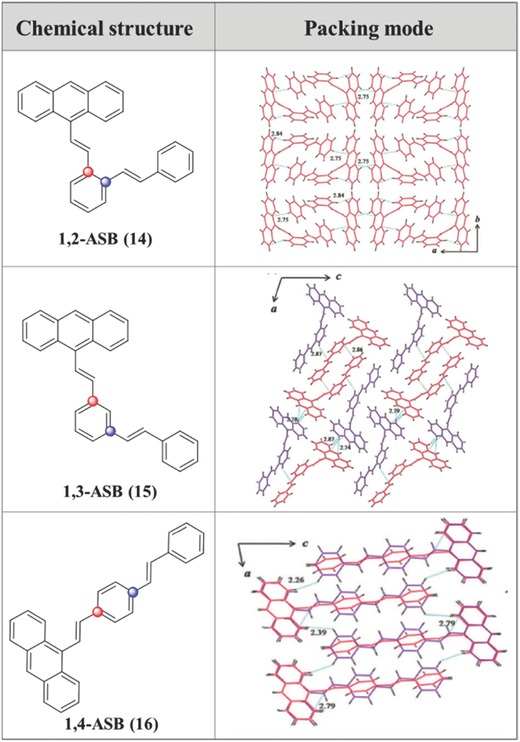
Chemical structures and packing modes of **14**–**16**.

Apart from the common TPE moiety, strong deep‐blue materials based on polyphenylbenzene, with highly twisted conformation, have also been obtained by a new designed synthetic approach and tunable linkage modes in our group.^50^ Following our proposed synthetic route, Jux and co‐workers synthesized a series of polyphenylbenzenes.^54^ Similarly, as shown in **Figure** **10**, in three (9‐anthryl)vinylstyrylbenzene position isomers, **14** and **15** demonstrate ACQ properties, while **16** is an AIEgen by the formation of crystal, which is also named crystallization‐induced emission.^55^ Compounds **14** and **15** with *ortho*‐ and *meta*‐linkage modes respectively, exhibit twisted structures, in which the anthryl group is almost vertical to the central benzene ring. The adjacent molecules are inclined to the edge‐to‐face packing modes with weak C—H⋯π interactions between benzene and anthracene rings. However, in the crystal structure of **16**, the dihedral angle between the anthryl moiety and benzene ring becomes small. A lamellar structure is formed with the parallel arrangement of adjacent anthracene rings, resulting in the strong π–π interactions with a shortest distance of 3.481 Å. This more closely packed structure can inhibit the intramolecular motion effectively, along with the decrease of nonradiative decay process. Thus, AIE is active by the subtle change of the linkage modes, coupled with the derived alignment of anthracene. Probably, it also indicates that the π–π stacking is not always bad to the AIE property.

**Figure 11:**
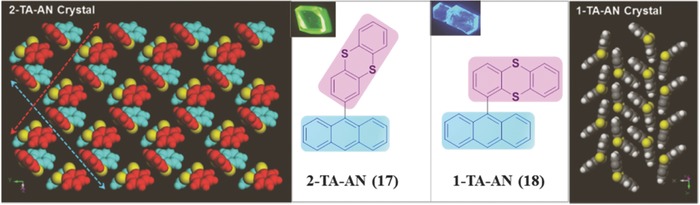
Chemical structures of **17** and **18** and the arrangement of thianthrene layer in their crystals.

Actually, the anthracene ring has been considered as one of the best candidates to study the relationship between intermolecular arrangement and fluorescence properties, with great attentions being attracted on the excimer formation of its derivatives.^56^, ^57^, ^58^ According to different packing modes, the emission color and fluorescent quantum yields varied in a large range. In compound **17**, 2‐(anthracen‐9‐yl)thianthrene, with the 2‐position linkage of thianthrene, the anthracene groups stack pairwisely with a typical face‐to‐face structure, and each anthracene dimer is spatially separated from the neighboring ones by bending thianthrenes (**Figure** **11**).^59^ This packing mode contributes to the restriction of nonradiative deactivation, and disables the photodimerization of anthracenes, then resulting in an unexpected high Φ_F_ (up to 80%) and long lifetime (163.75 ns) of the formed excimers, which should be ascribed to the symmetrically forbidden radiative transition from the excimer state to the ground state and the significantly suppressed nonradiative pathway. The corresponding fluorescence peak located at about 530 nm with green emission, mainly due to the large overlap of anthracene moieties. However, once the linkage position changed, compound **18** possesses a herringbone arrangement in its crystal, and the anthracene moieties stack in an antiparallel alignment. Accordingly, the Φ_F_ value is only 40%, with the emission in blue region.

**Figure 12:**
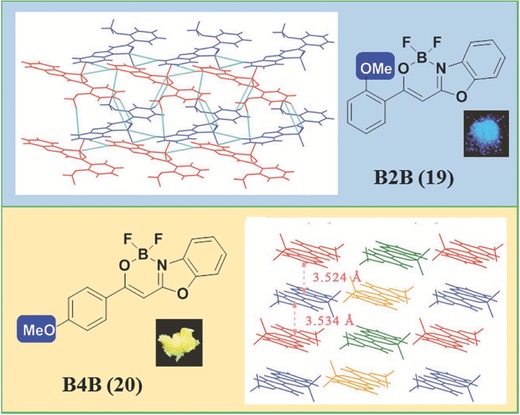
Chemical and crystal structures of **19** and **20**.

Besides the different linkage positions between aromatic rings, small nonaromatic groups can also affect the solid‐state emission.^60^ As shown in **Figure** **12**, compounds **19** and **20** have similar structures but different positions of the methoxyl group, which causes nearly no influence on the absorption and emission in solution, but the much different molecular packing in solid states.^61^ As the result, **19** emits blue light centered at 461 nm in single crystal, while a yellow emission with two peaks at 480 and 550 nm for **20**. Analyzing the crystal structure carefully, for **19**, the molecules (drawn in red and in blue) stack into two kinds of row‐liked structures in parallel to each other. Thus, the π–π stacking should be very weak, because of the small molecular overlapping degree with large dihedral angles between adjacent molecules. However, in the crystal of **20**, two antiparallel molecules stack into a dimer, and then into a column, with the distances between the adjacent molecules in a dimer and between the adjacent dimers as 3.524 and 3.534 Å, respectively.

**Figure 13:**
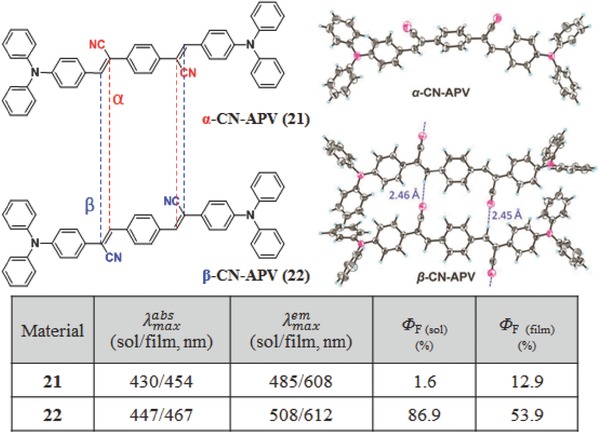
Chemical and crystal structures and optical properties of **21** and **22**.

In addition, the cyano unit as the electron‐withdrawing moiety has been introduced into the conjugated system frequently, to increase the ICT effect, and/or induce the mechanochromic and electrochromic properties, and/or arrange the packing modes by the well‐organized C—H⋯N interactions in different types.^62^, ^63^, ^64^ Taking the common distyrylbenzene derivatives as examples, once the cyano unit linked to the conjugated skeleton with different positions and spatial orientation, the resultant **21** and **22**, with similar structures, exhibit different optoelectronic properties (**Figure** **13**).^65^ The single crystal structure shows that **21** has a twisted backbone with the cyan groups on the same side. The torsional angles between the vinylene groups and their adjacent phenylene rings are 23° and 24°, which may promote the nonradiative decay process through the intramolecular motions. Thus, the Φ_F_ of **21** in solution is rather low (1.6%) with the maximum emission wavelength of 485 nm. However, in thin film, Φ_F_ can increase to 12.9%, since the vibration freedom of the CN groups could be limited by the weak intermolecular interactions. Thus, **21** is a typical AIE molecule. In contrast, **22** shows more planar backbones with smaller torsional angels (12.3° and 14.1°) between vinylene groups and phenyl rings. This planar configuration is beneficial to the intramolecular charge transfer between the electron donor (triphenylamine) and electron acceptor (CN), resulting in the red‐shifted absorption and emission spectra. Accordingly, the high Φ_F_ in solution (86.9%) is achieved for the stable excited state. In the crystal structure, the strong interactions between the two cyano and vinyl groups can be observed with tight intermolecular stackings, and the Φ_F_ in film decreases to 53.9%, exhibiting the ACQ property in some degree. But it is still higher than that of the AIE one (**21**, 12.9%), indicating that the resultant light‐emission properties of fluorescence molecules in the solid state not only depend on the fluorescent changes from single molecule to aggregated state (ACQ or AIE), but also the electron properties of the molecules themselves. Also, **22** demonstrates better hybrid local and charge transfer (HLCT) excited states, which is beneficial to its application in OLEDs.

The above examples proved that the different linkage positions of the substituent moieties can change the electronic properties and spatial configurations of the corresponding molecules, also, the different packing modes can be formed in accordance with various intermolecular interactions, resulting in the different light‐emission properties in the solid state. Normally, the planar conformation can benefit the intramolecular charge transfer, and the suitable intermolecular interactions and the uniform alignment can suppress the nonradiation process in the excited state. The balance of the two effects can yield the strong fluorescence in the solid state.

### 2.3 The Changes of Packing Modes by the Different Electronic Property, Chirality, and H‐bonding, as well as the Environmental Effect

**Figure 14:**
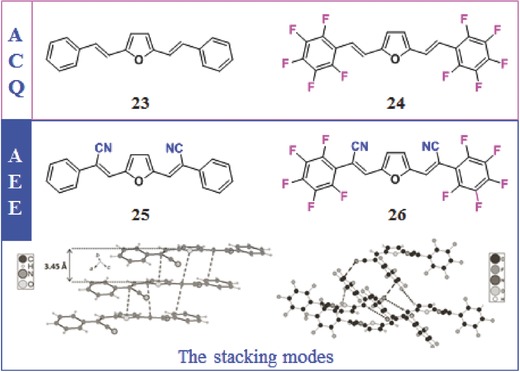
Chemical structures of **23**–**26** and the stacking modes of **25** and **26** in the crystal. **AEE**: aggregation‐enhanced emission.

The intermolecular interactions and packing modes of conjugated luminogens are complicated, which are influenced by some other factors, such as the electronic properties of aromatic rings, chirality, H‐bonding, and solvent effects, in addition to the above ones. Back to the frequently used benzene ring, its quadrupole moment will inverse, once its hydrogen atoms are replaced by fluorine ones, for their high electronegativity. The electrostatic quadrupole–quadrupole interactions between aryl (Ar) and perfluoroaryl (Ar_F_) can result in the well‐organized π‐stacks with alternating component molecules, meaning that the electronic properties of the aromatic rings can affect the packing modes in a large degree.^66^, ^67^ Particularly, the electron‐deficient aromatic rings interact most strongly with electron‐rich aromatic groups. Also, the additional electron‐donating or withdrawing moieties linked to the conjugated skeleton are liable to change the configurations and interactions of the corresponding molecules. As shown in **Figure** **14**, 23 and 24 with planar structures exhibit ACQ properties for the strong π–π stacking interactions. Upon the incorporation of CN unit, the opposite AIE activities of the resultant molecules (**25** and **26**) are apparent, mainly due to the slightly distorted configurations with the cyano groups twisted from the plane (defined by the furan ring).^68^ Meanwhile, in the aggregated state, the intermolecular CN⋯Ar_F_ and C—F⋯Ar_F_ interactions and hydrogen bonds rigidify the configuration largely, directly contributing to the observed AIE phenomena.

**Figure 15:**
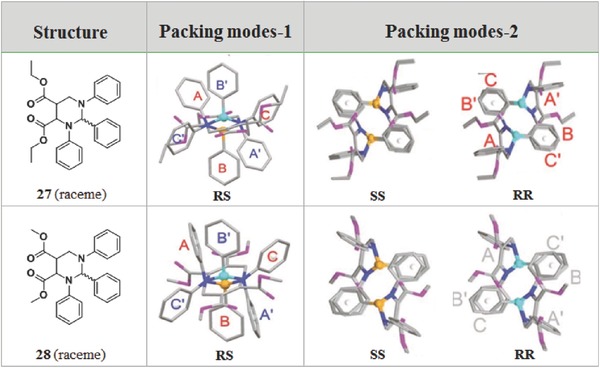
Chemical structures of **27** and **28** and two kinds of packing modes in the crystal with different chirality.

In spite of the extremely similar structures, for example, configurational isomerism, enantiomers with the mirror symmetry can form the different packing modes by various electron couplings.^69^ The structure characteristic of terahydropyrimidine derivatives is a nonaromatic chiral central ring connected with three aryl rings, which are not conjugated with each other. Thus, **27** and **28** show practically no emission in solution for the low conjugated molecular structures and high intramolecular motions, but strong emission in aggregates owing to the interesting RS‐ and RR/SS‐packing modes (**Figure** **15**). In a packing mode of R‐ and S‐enantiomers (RS‐packing mode), the paired enantiomers arranged as flowers with six petals (six phenyls) and more twisted conformations, while R‐ and S‐enantiomers self‐assemble as unpaired zigzag lines in the RR/SS‐packing mode. A less twisted conformation with the through‐space conjugation between the dicarboxylates is formed, accompanying with the strong AIE effect with Φ_F_ in crystal up to 93%. It suggests that the enhanced electron coupling and intramolecular charge transfer are also beneficial to the improvement of AIE property.

**Figure 16:**
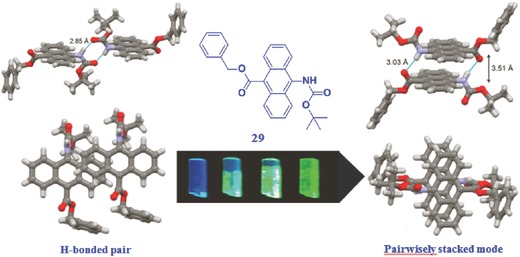
Chemical structures of **29** and two packing modes in crystal structures, blue dotted lines indicate H‐bonding associated with the N—O atomic distances.

Apart from the aromatic–aromatic interactions, the hydrogen bonding also plays an important role in the stacking and supramolecular chemistry, which can result in the formation of 1D chains or tapes, 2D sheets, and 3D structures.^70^, ^71^, ^72^ Both face‐to‐face arene–arene interactions and H‐bonding can contribute to the stability of the uncommon bilayer structure, which, in an isolated manner, could avoid the quenching effect in a large degree and possibly enhance their dimer emission. Taking an anthracene derivative as the example (compound **29** in **Figure** **16**), the construction of a pairwise packing of anthracene moieties is realized by introducing two H‐bonding sites, H‐bond donor and acceptor sites, at its 9‐ and 10‐positions, respectively. It is nonluminescent in ethanol solution, but shows the AIE activity in aggregate state. Fortunately, two polymorphic crystals of compound **29** with H‐bonded pair and pairwisely stacked mode, have been cultured with blue and green fluorescence, respectively.^73^ The different emission color is mainly caused by the different overlapping degrees of anthracene moieties. More interestingly, the blue emission can be changed to the green one quickly by just heating, due to the transformation from the monomer fluorescence to the excimer emission, as the result of the changed packing mode. Thus, the introduction of the hydrogen bonding is an efficient approach to realize the various artificial systems with different optoelectronic properties.

**Figure 17:**
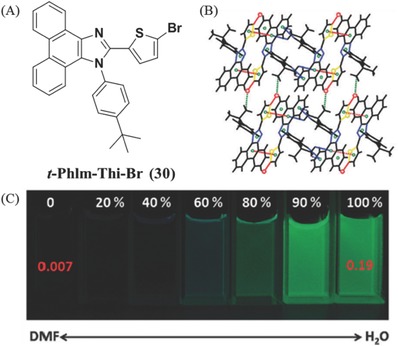
A) Chemical structure of **30**, B) the packing mode in the crystal, and C) its photographs in the mixtures of DMF and water (water contents 0%–100%) taken under 365 nm hand‐lamp irradiation.

Halogen atoms can also contribute much to the molecular packing sometimes with interesting luminescent phenomena, due to their heavy atom effect and possible presence of C—H⋯X (X = halogen) interactions. As shown in **Figure** **17**, in dimethylformamide (DMF) compound **30** is almost nonemissive with a Φ_F_ of 0.007, whereas it turns to green‐yellow emission with Φ_F_ up to 0.19 in pure water.^74^ However, if the bromine atom is replaced by the hydrogen one, the resultant molecule exhibits the ACQ property, and the strong emission in DMF is blue‐shifted largely in comparison with that of **30**, indicating the key role of halogen atom. In the crystal structure of **30**, two adjacent molecules are densely packed as a dimer by π–π interactions (3.59 Å) between the thiophene ring from one molecule and the phenanthrene ring from another in a head‐to‐tail fashion. C—H⋯Br interactions (3.08 Å) exist between two adjacent rows. The formation of J‐aggregate can strength the electron coupling of the adjacent molecules, leading to the enhanced emission and red‐shift of fluorescent spectrum, which can also be observed in many other AIE cases.^75^, ^76^, ^77^

**Figure 18:**
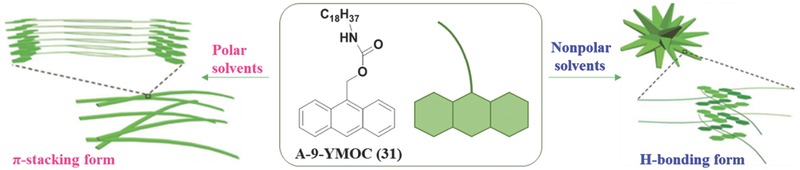
Chemical structures of **31** and schematic representation of its self‐assembled architectures in various solvents.

Except the influence of the molecular structure itself on the packing mode, in some case, the media exhibit the crucial impact. For example, the characteristics of solvents, such as the polarity, viscosity, hydrophilia, or hydrophobicity, can tune the self‐assembled architectures in the aggregated state, mainly due to the variation of noncovalent interactions between solute–solvent molecules.^78^ **Figure** **18** shows that the nanofibers and nanoflowers can be formed by the same compound of **31** in solvents with different polarities. In **31**, there are an anthracene head group, an imide group, and a long alkyl chain, which offer the liability for the π–π stacking, hydrogen bonding, and dispersion interactions, respectively. In polar solvents, the hydrogen bonding of amide groups is the main driving forces for the molecular assembly, resulting in the outside packed polar groups and the interior of bilayer arrays from the long alkyl chains, to avoid the solvent molecules with high polarity. Conversely, in nonpolar solvents, it tends to form tetragonal columnar structures, in which, the alkyl chains extend outside and the polar groups remain inside, inherently forced by the π–π stacking and dispersion interactions. Thus, it is excited but not surprising that the transition of morphology from nanofibers into nanoflowers can be achieved by choosing appropriate solvents, providing a simple way to manipulate the morphology and properties of self‐assembled organic building blocks.

For the organic luminogens based on π‐system, as mentioned above, most of the AIE activities can be derived from two ways:

(a) From the molecule itself with twisted conformation. The formation of aggregated state, either nanoparticle or solid state, can inhibit the possible intramolecular motions in solution by the stacking modes or intermolecular interactions.
(b) The electron coupling of the adjacent molecules by J‐aggregation, H‐aggregation, and the excimer formation. In this case, usually, the fluorescent emission is different from that of the single molecules.

**Figure 19:**
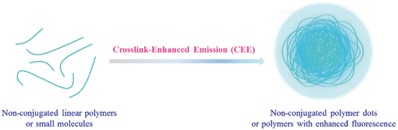
Representation of crosslink‐enhanced emission (CEE) in nonconjugated polymer dots or polymers.

Interestingly and excitedly, similar phenomena can also be observed in nonconjugated systems (**Figure** **19**).^79^, ^80^ Generally, the nonconjugated linear polymers or small molecules only possessing subfluorophores (which are groups such as C=O, C=N, N=O) instead of typical conjugated fluorophore groups, should not demonstrate strong fluorescence in the usual sense. However, after the efficient immobilization routes consisted of covalent bond, supramolecular interaction, and rigidity aggregation, the vibration and rotation of the subfluorophore are restricted, leading to an increase of radiative transition, possibly accompanying with the new formed molecular energy levels. For example, once the polyvinyl alcohol (PVA) aqueous solution is frozen to form a gel, the fluorescence appears.^81^ Also, the hydrothermal treatments can crosslink PVA partially to form the cross‐linked dots with the confined oxygen‐based subfluorophores, as a result, the blue emission can be observed. Different from the fluorescence of conjugated system, the fluorescence of nonconjugated polymer dots (NCPDs) is dependent on the excitation wavelength (λ_ex_), indicating that there are multiple excited states perhaps without energy transfer between them.

## 3 The Organic Molecules with RTP

**Figure 20:**
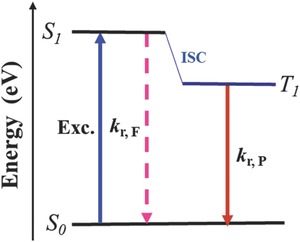
Energy‐level diagram of the relevant photophysical processes in RTP materials.

Unlike fluorescence, phosphorescence is a spin‐forbidden radiative transition from the lowest triplet excited state (T_1_) to the ground state (S_0_) (**Figure** **20**). Basically, without precious metals, the rate of phosphorescence is very slow, thus, the RTP of pure organic molecules is hardly observed, mostly due to the presence of triplet oxygen, thermal and vibrational relaxation, inaccessible triplet states, weak spin–orbit coupling, and highly forbidden triplet–singlet transitions. Excitedly, in recent years, thanks to the well‐organized alignments of molecules in the solid state, some pure organic RTP luminogens with relatively long lifetimes have been reported.^82^, ^83^, ^84^, ^85^, ^86^

**Figure 21:**
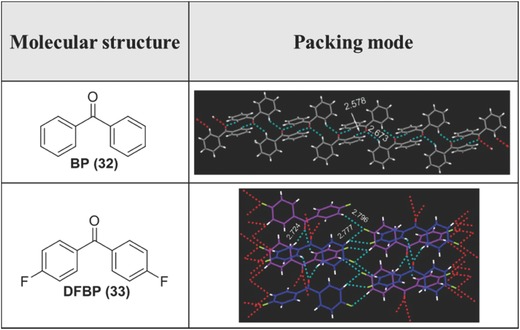
Chemical structures and packing modes of compounds **32** and **33**, intermolecular interactions are marked by the dotted lines.

In 2010, Tang and co‐workers reported an unprecedented observation of highly efficient RTP from a large variety of organic carbonyl and halo‐arenes.^87^ Taking the simple compound **32** (**Figure** **21**) as an example, it does not show any room‐temperature emission in solution or aggregated states. But its crystal shows RTP around 420–480 nm with a quantum yield of phosphorescence (Φ_ph_) as high as 15.9%, indicating that nonradiative deactivation of triplet states in solution states can be suppressed by intermolecular interactions of the C—H⋯O hydrogen bonds. After the introduction of two fluorine atoms at the 4,4′‐positions of BP, compound **33** exhibits stronger phosphorescence with a Φ_ph_ of 39.7%, and the lifetime is as long as 1296.9 µs. As shown in Figure 21, the additional C—H⋯F interactions are involved in the packing arrangement of the crystal, which can further enhance the phosphorescence by suppressing the energy loss through the thermal relaxation processes.

Generally, with a lone pair of electrons on the oxygen atom, the aromatic carbonyl possesses a degree of spin–orbit coupling, which allows for the intrinsic triplet generation from S_1_ to T_1_ through the intersystem crossing (ISC). The present halogen atom is helpful to lock the molecular conformation for the formation of rigid crystal packing via the halogen bonding. Also, the heavy atom effect can promote the singlet–triplet conversion. Both of these two compositions are beneficial to the existence of RTP.

**Figure 22:**
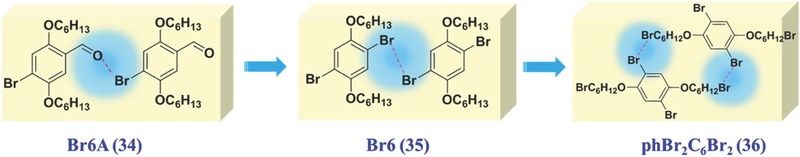
Chemical structures of compounds **34–36** and heavy‐atom interaction in the solid state.

Subsequently, a large number of examples of pure organic phosphorescent luminogens were reported, based on crystal engineering via the manipulation of weak hydrogen and halogen bonding interactions in the solid state.^88^, ^89^, ^90^ When the molecule consists of both aromatic aldehyde and bromine atoms, the latter can interact with the oxygen atom of the aldehyde group in another neighbor molecule through a noncovalent approach, partially delocalizing the oxygen electrons onto the bromine one. This halogen bonding of C=O⋯Br with the O⋯Br distance of 2.86 Å (**Figure** **22**), could direct the heavy atom effect to the active triplet site of compound **34**, enhance the intersystem crossing, and reduce the vibrational losses at the carbonyl to activate the phosphorescent emission.^91^ Thus, under excitation at 360 nm, **34** emits a green phosphorescence at 500 nm in the solid state, totally different from its weak fluorescence in solution. However, due to the close spacing of the molecules, a Φ_ph_ of 2.9% is obtained, mainly due to the self‐quenching processes in crystal. To avoid this quenching, **34** was dispersed in the analogue host of **35** by introducing additional bromine atom instead of the carbonyl one, to form guest–host systems with the diluted concentration of **34**. By tuning the ratio of **34** and **35** with the varied electron density and triplet level, the mixed crystals of **34**/**35** exhibit different emission colors, with the maximum Φ_ph_ of 55% and lifetime of 8.3 ms. Due to the advantage of the heavy atom effect, the manipulation of heavy‐atom interaction is also conducted by replacing alkyl terminal groups of **35** with bromine atoms. And the designed molecule of **36** demonstrates high Φ_ph_ up to 21.9% and millisecond‐scale lifetime, indicating that the phosphorescence generation could be promoted by increasing the bromine‐cluster interactions and decreasing the triplet–triplet annihilation chance.^92^ Also, some other examples can further confirm the important role of halogen bonds, such as the green phosphorescence observed from the crystal of a bromosubstituted capped γ‐amino acid foldamer.^93^ Interestingly, other moieties, including siloxy groups, borylated tellurophenes, and sulfones, are also beneficial to RTP.^94^, ^95^, ^96^

**Figure 23:**
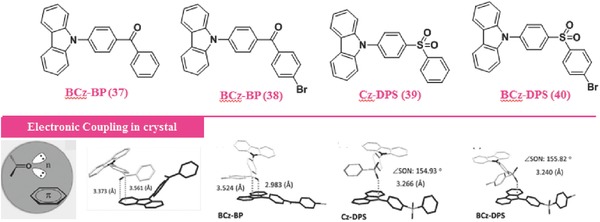
Chemical structures of **37**–**40** with room‐temperature phosphorescence and intermolecular electronic coupling in the crystal.

Recently, a new explanation for organic RTP is proposed that the bright long‐lived RTP, as the results of the formed hybrid ISC transitions, could be realized, by combining the high ISC and low radiative rates through the intermolecular electronical coupling of different groups containing the lone pair n‐electrons or π‐ones, in the photophysical processes of some crystals.^97^ For example, with a carbonyl or sulfonyl group as the unit bearing n‐electrons and a carbazolyl (Cz) moiety as the π‐one, RTP with remarkably long lifetimes (up to 0.28 s) and high quantum efficiency were observed in compounds **37**–**40** (**Figure** **23**), in which, the π‐unit also acts as an electron donor (D) and the n‐one as an acceptor (A). Because of the twisted configuration between D and A moieties, the spatial overlap between the highest occupied molecular orbital (HOMO) and lowest unoccupied molecular orbital (LUMO) is reduced, resulting in the small energy gap between the S_1_ and T_1_ excited states, leading to the increase of the ISC rate for phosphorescence. In crystals, the close intermolecular stacking is formed for the electrostatic interactions between D and A moieties. For instance, the carbonyl group in compound **37** stacks approximately parallel to the Cz group in a neighbor molecule, with the distance as short as 3.373 Å for the oxygen atom and 3.561 Å for the carbon atom to the Cz plane. This stacking mode really contributes much to the significant intermolecular interactions of their orbitals, thereby resulting in the intensive RTP.

**Figure 24:**
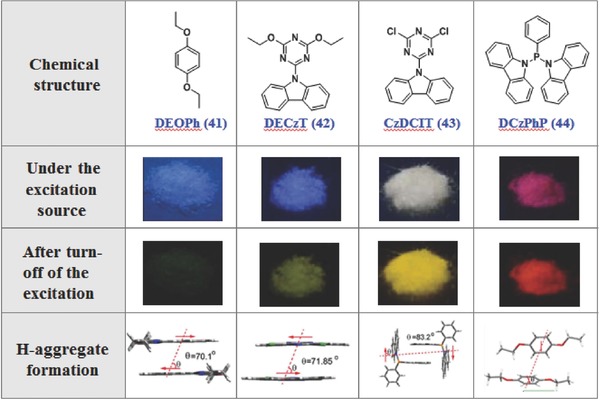
Chemical structures of **41–44** with ultralong room‐temperature phosphorescence and their packing modes in the crystal.

Besides the strong noncovalent interactions between special atoms, the aggregation forms, as discussed for AIEgens above, can also affect the production of triplet excitons, possibly leading to the RTP luminogens. Chen, Liu and Huang groups reported some excited luminogens of this kind (**Figure** **24**), which packs in the H‐form with the promotion to produce triplet excitons through the newly formed triplet excited state (T_1_*) after photoexcitation followed by the intersystem crossing.^98^ For compound **42**, the phosphorescence lifetime was measured to be as long as 1.35 s, which is several orders of magnitude longer than those of conventional organic fluorophores.

## 4 Conclusions

In this paper, based on some selected examples and the hidden thoughts of the authors, we try to disclose the influence of the molecular packing status, in addition to the electronic structures, on their emissive properties in the aggregated state. Mainly, two kinds of luminogens are involved, pure organic AIEgens and RTP luminogens, for their abnormal and shining characteristics. All the elaborately chosen cases exhibit the key role of molecular packing in aggregated state, often leading to the much different emissive behavior from that in solution. Taking AIEgens as examples, the dramatically enhanced emission in the aggregated state, in some degree, subverts the traditional thought lost in the concentration quenching effect, and arouses the deep thinking of solid‐state emission, providing an alternative approach to develop strong emitters in solid state, in addition to the conventional strategies against aggregation. Actually, in pursue of good luminogens for practical applications, the essential point is the high emissive efficiency in aggregated state, but not whether with AIE characteristic or not, just as the cases of **21** and **22**. However, in some cases like sensors, the AIE characteristic, especially nonemissive in solution but emissive in aggregate state, possesses inherent and unique advantages. Undoubtedly, the packing status affects the emission largely, even with the formation of excimer for pyrene. However, the previous summarized design principles for luminogens, perhaps, have emphasized heavily on their electronic properties, ignoring, in a large degree, the packing status in aggregates and the possibly new born energy levels. Thus, the present structure–property relationships focus mainly on the structure of single molecule and the performance of molecular aggregates, lacking the important information of molecular packing. As partially demonstrated in this review paper, scientists have strengthened the researches on how packing status affecting the performance. However, for the further and rapid development of the investigation on the packing mode, perhaps, at least three issues should be considered seriously: the defined structure with packing status, the related theory, and the accurate structure–packing–performance relationship.

In all the above cases, the packing status is well illustrated by the single crystal structure, without which, no explanations could be proposed for the observed interesting and excited phenomena. But, regardless of the present powerful single‐crystal diffratometer and the culture technique, it is still a lucky thing to get a crystal with high quality, especially for compounds with polymorphism. As to polymers, the crystal culture, perhaps, is an nearly impossible task. Thus, how to collect the accurate information of the packed molecules, should be a challenge, not only limited to the material science.

In functional materials, especially for those based on π‐conjugated systems, the theoretical calculations, generally the electronic cloud distribution on HOMO and LUMO orbitals, can be much helpful to explain and understand the obtained experimental results. Also, we could conduct other related calculations based on the well‐established theories for single crystals. However, there are still huge room for the developments of the related theories focusing on the molecular aggregates, to understand the energy levels of new prepared functional materials, such as nonconjugated polymer dots shown in Figure 19, and the widely investigated carbon dots with strong emissions.

The third one, but not the last one, is the accurate structure–packing–performance relationship, which needs so many examples, related information, rational analysis, and deep thinking. Not easy, but so important! It requires the cooperation of scientists in different research fields. Actually, the relationship is not only applicable to AIEgens and RTP luminogens discussed in this paper, but also widely useful to other functional materials, for example, those utilized in the hot topics of polymeric photovoltaic cells and field‐effect transistors, in which, the control of the molecular packing is the key point to achieve high efficiency.

So far, some feelings have been sensed and hidden in scattered literatures, accompanying with the deep investigation as inherently required from scientists themselves, the reliable rules could be constructed. Perhaps, just like the new excited properties aroused by the special packing of single molecules, the “packing” of scientists with different backgrounds can yield wonderful new recognitions.
